# 16322 Post-Hurricane Community Health Assessment through Newspaper Stories and Interprofessional Community Engagement

**DOI:** 10.1017/cts.2021.607

**Published:** 2021-03-30

**Authors:** Kathleen R. Stevens, Mary Judson, Dan Parker, Bridgett Piernik-Yoder, Wendy Lee, Timothy Reistetter, David Vasquez

**Affiliations:** 1 University of Texas Health San Antonio; 2 South Jetty Newspaper, Port Aransas, TX; 3 Texas Department of Health and Human Services, San Antonio, TX

## Abstract

ABSTRACT IMPACT: Working alongside news staff as community partners is feasible for community engagement to co-create a post-hurricane health assessment and connect it to our academic health center’s disaster response capacity. OBJECTIVES/GOALS: Successful academic-community partnership in post-disaster response depends on shared understanding of impact. Community newspapers could provide valuable insight into health needs and inform strategic recovery plans. Our objective was to determine methodological feasibility of using newspaper stories to identify post-disaster needs. METHODS/STUDY POPULATION: Community-Based Participatory Research principles were applied to engage newspaper staff and conduct qualitative analysis of stories published in the weekly Port Aransas South Jetty newspaper, serving this small rural coastal community. Using directed content analysis, the team derived and validated constructs from Maslow’s Hierarchy of Needs and Phases of Disaster models to create a codebook. Scientists and newspaper staff examined the codebook for congruency regarding interpretation and themes. With copyright permission to access online newspaper files, NVivo software was used to search for Hurricane Harvey-related terms (e.g., ‘Harvey, tropical storm, flood, damage, volunteer’). Stories from 3 days post-Harvey to 6 months post-Harvey were examined and again at anniversary date. RESULTS/ANTICIPATED RESULTS: The weekly South Jetty newspaper was published continuously from August 31, 2017, through the date our study ended, February 22, 2018. Analysis showed themes of the storm and community response to disaster at multiple levels. Harvey caused catastrophic flooding, destruction, on par with 2005 Hurricane Katrina as the costliest storm on record. In Port Aransas, 130 mph winds and a 12-foot storm surge damaged 90% of the buildings. Stories reflected Phases of Response: Pre-disaster, Impact, Heroic, Honeymoon, Disillusionment, and initial phases of Reconstruction and Maslow’s Hierarchy of Needs. Story: ‘It’s not just the physical part of Port Aransas that was hurt by the hurricane. Harvey also wounded the town’s collective psyche. We’ve wept for our losses, then counted our blessings, then wept for our losses again.’ DISCUSSION/SIGNIFICANCE OF FINDINGS: Newspapers were a rich source of post-disaster data. Text and pictures were poignant. Thematic analysis identified stages of recovery. Working alongside news staff as community partners is feasible for community engagement to co-create a post-hurricane health assessment and connect it to our academic health center’s disaster response capacity.

